# A nomogram based on platelet-to-lymphocyte ratio for predicting lymph node metastasis in patients with early gastric cancer

**DOI:** 10.3389/fonc.2023.1201499

**Published:** 2023-08-31

**Authors:** Hongyu Wu, Wen Liu, Minyue Yin, Lu Liu, Shuting Qu, Wei Xu, Chunfang Xu

**Affiliations:** Department of Gastroenterology, The First Affiliated Hospital of Soochow University, Suzhou, China

**Keywords:** early gastric cancer, lymph node metastasis, platelet-to-lymphocyte ratio, prediction model, nomogram

## Abstract

**Background:**

Preoperative assessment of the presence of lymph node metastasis (LNM) in patients with early gastric cancer (EGC) remains difficult. We aimed to develop a practical prediction model based on preoperative pathological data and inflammatory or nutrition-related indicators.

**Methods:**

This study retrospectively analyzed the clinicopathological characteristics of 1,061 patients with EGC who were randomly divided into the training set and validation set at a ratio of 7:3. In the training set, we introduced the least absolute selection and shrinkage operator (LASSO) algorithm and multivariate logistic regression to identify independent risk factors and construct the nomogram. Both internal validation and external validation were performed by the area under the receiver operating characteristic curve (AUC), C-index, calibration curve, and decision curve analysis (DCA).

**Results:**

LNM occurred in 162 of 1,061 patients, and the rate of LNM was 15.27%. In the training set, four variables proved to be independent risk factors (p < 0.05) and were incorporated into the final model, including depth of invasion, tumor size, degree of differentiation, and platelet-to-lymphocyte ratio (PLR). The AUC values were 0.775 and 0.792 for the training and validation groups, respectively. Both calibration curves showed great consistency in the predictive and actual values. The Hosmer–Lemeshow (H-L) test was carried out in two cohorts, showing excellent performance with p-value >0.05 (0.684422, 0.7403046). Decision curve analysis demonstrated a good clinical benefit in the respective set.

**Conclusion:**

We established a preoperative nomogram including depth of invasion, tumor size, degree of differentiation, and PLR to predict LNM in EGC patients and achieved a good performance.

## 1 Introduction

Until 2020, gastric cancer (GC) has ranked fifth for incidence and fourth for mortality worldwide (1); in the meanwhile, it is the second leading cause of cancer-related deaths in China (2). Gastric cancer can be classified as early gastric cancer (EGC) and advanced gastric cancer (AGC) clinically, according to the definition proposed by Japan Gastric Cancer Association (JGCA), that cancerous lesions are confined to the mucosa or submucosa without regard to the status of lymph node metastasis (LNM) (3, 4). In general, patients diagnosed with early gastric cancer had favorable prognoses; however, once lymph node metastasis took place, the long-term survival prognosis declined drastically (5).

In recent years, the rapid development of endoscopic technologies has considerably increased the detection rate of early gastric cancer (6), improving the survival prognosis of patients with gastric cancer. The second edition guidelines for endoscopic submucosal dissection (ESD) and endoscopic mucosal resection (EMR) for EGC have been applied widely and continuously expanding the indications (7). However, since minimally invasive endoscopic surgery cannot entirely remove local lymph nodes, total or subtotal gastrectomy with systematic D1+/D2 lymphadenectomy is still imperative for patients with LNM (8–11). Therefore, the prediction of lymph node metastasis in EGC patients is of particular significance in determining the best treatment option in clinical practice to maximize benefit.

Until now, preoperative assessment of the presence of LNM in EGC patients has remained difficult. Although previous studies attempted to establish nomograms to predict lymph node metastasis, the majority of these models included lymphovascular invasion as an independent risk factor (12–17), regardless of the fact that it cannot be detected accurately without postoperative pathologic findings. The eCura system, a comparatively authoritative risk assessment system proposed by JGCA, evaluates the risk of LNM in post-ESD patients based on five factors: lymph invasion, tumor size, vertical margin, venous invasion, and submucosal invasion degree (18, 19). Furthermore, it determines whether follow-up surgical operations are needed according to the grade (7, 20, 21). The obvious drawback, in turn, is that it is performed in patients who have already undergone ESD, while additional surgeries usually result in additional risks.

Noteworthy, more attention has been paid to inflammatory biomarkers in various malignancies. To date, several indicators of inflammation or nutritional status in the peripheral blood, such as albumin, neutrophil-to-lymphocyte ratio (NLR), platelet-to-lymphocyte ratio (PLR), monocyte-to-lymphocyte ratio (MLR), and fibrinogen-to-albumin ratio (FAR), are thought to be useful prognostic biomarkers for gastric cancer, pharyngeal cancer, ovarian cancer, lung cancer, and esophageal cancer (22–26). We select these indicators as potential risk factors in that they are calculated by routine blood tests at admission. It means that they are easily acquired, and patients do not have to pay for extra examinations. In consequence, we aim to develop a practical prediction model based on preoperative pathological data and accessible inflammatory or nutrition-related indicators in the peripheral blood and to evaluate its reliability and clinical effectiveness.

## 2 Materials and methods

### 2.1 Patients

**Figure 1:**
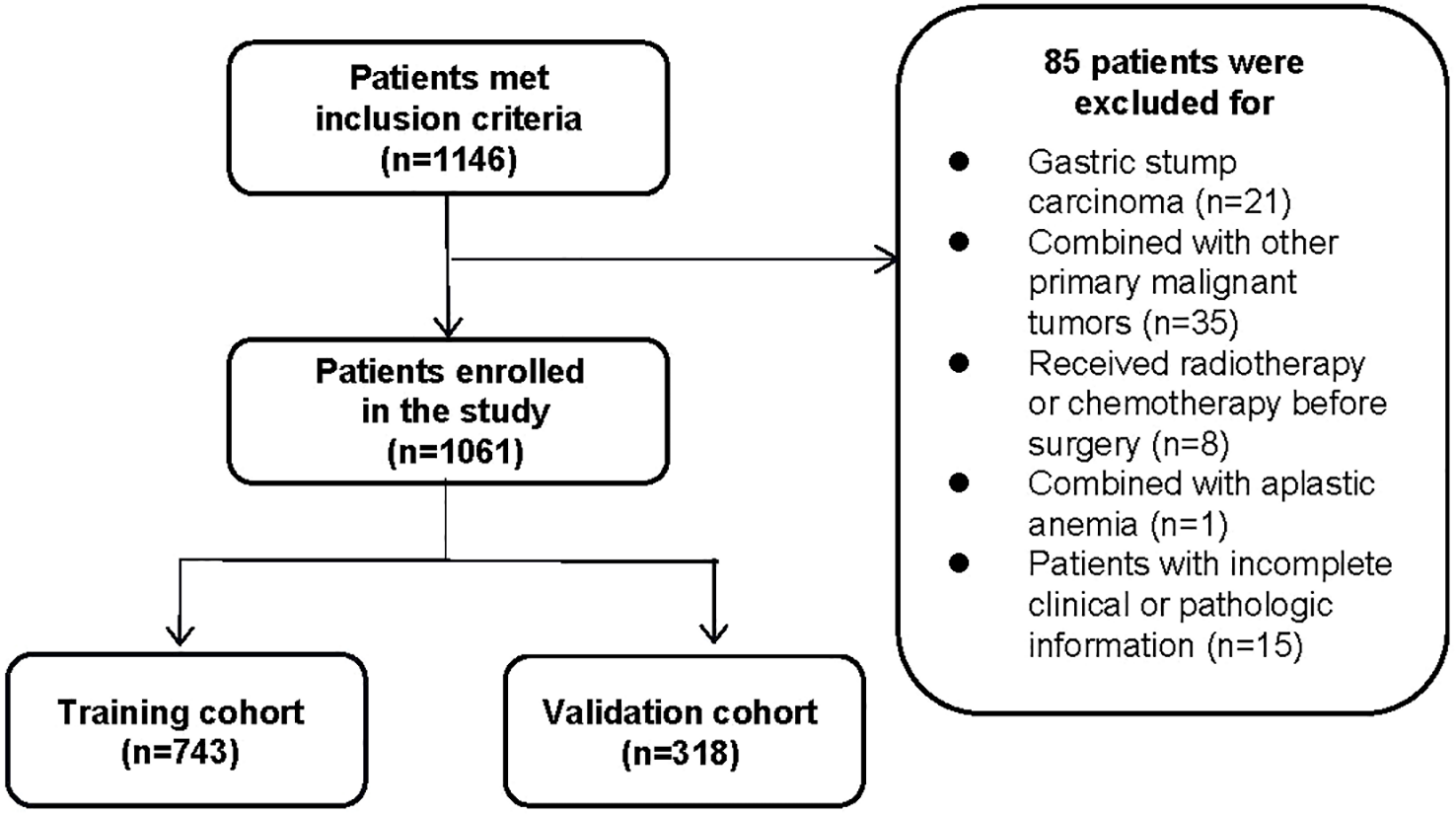
The flowchart of inclusion and randomization.

Clinical and pathological data on patients who underwent radical gastrectomy with D1/D1 + or D2 lymphadenectomy at the First Affiliated Hospital of Soochow University and were diagnosed with early gastric cancer by postoperative pathology were collected retrospectively from January 2016 to December 2021. A total of 1,146 cases were included, with 85 excluded for meeting the corresponding exclusion criteria (**Figure 1**), and 1,061 patients were eventually enrolled. The exclusion criteria were as follows: 1) patients with gastric stump carcinoma or recurrent gastric cancer; 2) combined with other primary malignant tumors; 3) have undergone preoperative chemotherapy, radiotherapy, or immunotherapy; 4) combined with severe liver/kidney diseases or other diseases may lead to hematologic abnormalities or primary hematologic diseases; 5) with incomplete clinical or pathologic information. Patients were divided into positive and negative groups for lymph node metastasis based on postoperative lymph node biopsies. This study was approved by the Ethics Committee of the First Affiliated Hospital of Soochow University. Since it was a retrospective analysis, informed consent from patients was not necessary.

### 2.2 Data collection

All the clinicopathological data were reviewed, including gender, age, body mass index (BMI), underlying diseases (presence or absence of hypertension/diabetes), tumor site, depth of invasion, lesion size, morphological characteristics, degree of differentiation, presence or absence of ulcer, and laboratory indicators including white blood cell (WBC) count, lymphocyte (LY) count, monocyte (MO) count, neutrophil (NE) count, platelet (PLT) count, the content of hemoglobin (HGB), fibrinogen, albumin (ALB), prealbumin (PAB), plasma total cholesterol (TC), triglyceride (TG), high-density lipoprotein cholesterol (HDL-C), low-density lipoprotein cholesterol (LDL-C), NLR, MLR, PLR, FAR, and fibrinogen-to-prealbumin ratio (FPR). BMI was divided into lean (<18.5 kg/m^2^), normal (18.5–24 kg/m^2^), and overweight (≥24 kg/m^2^). The tumor site was classified as cardia/fundus, gastric body, and antrum/pylorus of the stomach. The depth of invasion was classified as a lesion confined to the mucosa (pT1a) and submucosa (pT1b) according to the pathological biopsy. The lesion size was calculated according to the maximum diameter of the tumor in the pathological specimen, which was divided into the ≤2 cm group, 2–3 cm group, and >3 cm group. The degree of differentiation was classified as high, moderate, and poor differentiation. Gross type was classified as polypoid type, flat type, and excavated type according to the Paris classification (27). All enrolled patients underwent lymph node resection, and the total number of metastatic lymph nodes was reported. Blood samples were collected within 48 hours of admission.

### 2.3 Statistical analysis

Patients were randomly divided into training and validation sets in a 7:3 ratio. The model was developed in the training set and verified in the validation set. To reduce the effect of multicollinearity, the least absolute selection and shrinkage operator (LASSO) algorithm was introduced to screen the variables. The cross-validation method was used to determine the final lambda (λ). When lambda (λ) took the maximum value within one standard error of the minimum mean square error, the corresponding variables were taken into a multivariate logistic regression analysis to finally obtain variables with p-value <0.05 as independent risk factors. A nomogram was developed to visualize the results of the multivariate analysis. Simultaneously, receiver operating characteristic (ROC) curves were plotted, and the area under the ROC curve (AUC) (95% confidence interval) was calculated to quantify the discriminatory ability of the nomogram. An AUC of 1.0 would represent a perfect prediction, while 0.5 would represent a meaningless prediction. The concordance index (C-index) was used to evaluate the prediction accuracy of the model. In addition, the consistency between the predicted probability of LNM and the actual incidence was assessed by the calibration curve. Decision curve analysis was plotted to see the net benefit. Finally, internal validation was performed using bootstrap with 1,000 replicates, and external validation was performed in the validation set. All the statistical analyses were completed using R software (version 4.2.1). Data conforming to normal distribution were described as X ± SD in the numerical variables, while data conforming to abnormal distribution were described as median (quartile) [M (P25, P75)]. Categorical variables were measured by the chi-square test. A two-tailed p-value <0.05 was supposed to be significant.

## 3 Results

### 3.1 Baseline characteristics

According to the inclusion and exclusion criteria, 1,061 patients were included in the analysis, whose baseline characteristics are shown in **Table 1**. Lymph node metastasis occurred in 162 of 1,061 patients, and the rate of lymph node metastasis was 15.27%, which was consistent with some studies (28). Among them, 102 cases were in stage N1, 47 cases in stage N2, and 13 cases in stage N3. What is worth mentioning is that the rate of lymph node metastasis was 4.62% with lesions confined to the mucosa (476 cases), while the metastasis rate dramatically increased to 23.93% with lesions invading the submucosa (585 cases).

**Table 1:** Baseline characteristics of patients with early gastric cancer who underwent radical gastrectomy.

Clinicopathological parameters	Total number (%) /(mean ± SD)	LNM (n [%]) /(mean ± SD)	Non-LMN (n [%]) /(mean ± SD)
Hypertension			
Absent Present	688 (64.8) 373 (35.2)	109 (67.3) 53 (32.7)	579 (64.4) 320 (35.6)
Diabetes			
Absent Present	968 (91.2) 93 (8.8)	148 (91.4) 14 (8.6)	820 (91.2) 79 (8.8)
**Age (years)**	63.04 ± 10.48	62.41 ± 11.51	63.15 ± 10.29
Gender			
Male Female	733 (69.1) 328 (30.9)	103 (63.6) 59 (36.4)	630 (70.1) 269 (29.9)
BMI (kg/m^2^)			
≤18.5 18.5–24 >24	61 (5.7) 611 (57.6) 389 (36.7)	11 (6.8) 90 (55.6) 61 (37.7)	50 (5.6) 521 (58.0) 328 (36.5)
Depth of invasion			
Mucosa Submucosa	476 (44.9) 585 (55.1)	22 (13.6) 140 (86.4)	454 (50.5) 445 (49.5)
Differentiation			
Poorly Moderately Highly	550 (51.8) 355 (33.5) 156 (14.7)	114 (70.4) 45 (27.8) 3 (1.9)	436 (48.5) 310 (34.5) 153 (17.0)
Ulcer			
Absent Present	358 (33.7) 703 (66.3)	35 (21.6) 127 (78.4)	323 (35.9) 576 (64.1)
Diameter (cm)			
≤2 2–3 >3	616 (58.1) 257 (24.2) 188 (17.7)	62 (38.3) 53 (32.7) 47 (29.0)	554 (61.6) 204 (22.7) 141 (15.7)
Location			
Cardia/fundus Gastric body Antrum/pylorus	180 (17.0) 181 (17.0) 700 (66.0)	14 (8.6) 28 (17.3) 120 (74.1)	166 (18.5) 153 (17.0) 580 (64.5)
Paris classification			
Polyp Flat Depressed	79 (7.4) 533 (50.2) 449 (42.3)	16 (9.9) 53 (32.7) 93 (57.4)	63 (7.0) 480 (53.4) 356 (39.6)
**WBC (10^9^/L)**	5.69 ± 1.76	5.51 ± 1.84	5.72 ± 1.74
**LY (10^9^/L)**	1.70 ± 0.59	1.60 ± 0.53	1.72 ± 0.60
**MO (10^9^/L)**	0.41 ± 0.15	0.39 ± 0.14	0.41 ± 0.15
**NE (10^9^/L)**	3.43 ± 1.50	3.36 ± 1.67	3.44 ± 1.46
**PLT (10^9^/L)**	202.98 ± 65.57	215.05 ± 73.55	200.80 ± 63.83
**HGB (g/L)**	133.10 ± 20.56	130.08 ± 20.04	133.64 ± 20.62
**Fib (g/L)**	2.78 ± 0.73	2.78 ± 0.80	2.78 ± 0.72
**ALB (g/L)**	41.64 ± 4.90	40.77 ± 4.94	41.80 ± 4.88
**PAB (mg/L)**	227.43 ± 56.80	218.12 ± 53.69	229.10 ± 57.21
**TC (mmol/L)**	4.55 ± 0.97	4.56 ± 1.17	4.55 ± 0.93
**TG (mmol/L)**	1.70 ± 6.14	1.33 ± 0.76	1.76 ± 6.66
**HDL (mmol/L)**	1.58 ± 6.93	1.20 ± 0.32	1.65 ± 7.52
**LDL (mmol/L)**	2.87 ± 5.86	2.69 ± 0.79	2.90 ± 6.36
**NLR**	2.31 ± 1.86	2.45 ± 2.61	2.28 ± 1.70
**MLR**	0.26 ± 0.14	0.26 ± 0.12	0.26 ± 0.15
**PLR**	131.32 ± 61.84	150.04 ± 88.80	127.95 ± 55.00
**FPR**	13.49 ± 8.12	13.90 ± 6.96	13.42 ± 8.31
**FAR**	0.07 ± 0.03	0.07 ± 0.02	0.07 ± 0.03

BMI, body mass index; WBC, white blood cells; LY, lymphocytes; MO, monocytes; NE, neutrophils; PLT, platelets; HGB, hemoglobin; Fib, fibrinogen; ALB, albumin; PAB, prealbumin; TC, total cholesterol; TG, triglyceride; HDL-C, high-density lipoprotein cholesterol; LDL-C, low-density lipoprotein cholesterol; NLR, neutrophil-to-lymphocyte ratio; MLR, monocyte-to-lymphocyte ratio; PLR, platelet-to-lymphocyte ratio; FPR, fibrinogen-to-prealbumin ratio; FAR, fibrinogen-to-albumin ratio.

### 3.2 Screening of variables

**Figure 2:**
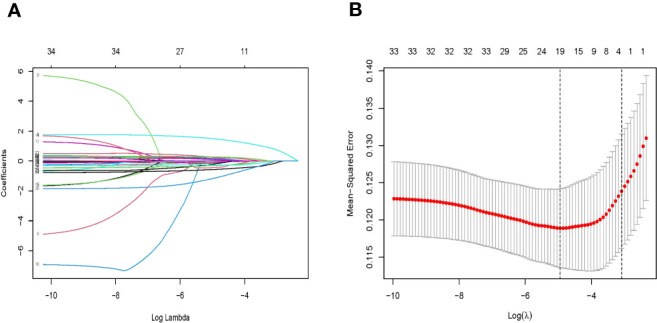
**(A)** Identification of the optimal penalization coefficient lambda (λ) in the LASSO model with 10-fold cross-validation in the training set. **(B)** LASSO coefficient profiles of 33 features. LASSO, least absolute selection and shrinkage operator.

In the training set, patients were divided into lymph node metastasis (+) group and non-lymph node metastasis (−) group based on postoperative pathological findings. The LASSO cross-validation was used to screen variables, and the results showed that four relevant factors with non-zero coefficients for EGC patients with LNM were screened at the optimum lambda (λ = 0.0456966, i.e., lambda.1se), including depth of invasion, tumor size, degree of differentiation, and platelet-to-lymphocyte ratio (**Figure 2**).

### 3.3 Multivariate analyses in the training set

The screened variables were subsequently included in a multivariate logistic analysis using backward stepwise regression to obtain the smallest Akaike information criterion (AIC) to improve conciseness and accuracy. Four variables proved to be independent risk factors (p < 0.05) and were included in the final model (**Table 2**).

**Table 2:** Multivariate analysis of independent risk factors in the training set.

	B	SE	OR	95%CI	Z	p
Depth of invasion						
Mucosal Submucosal	Ref 1.682	Ref 0.291	Ref 5.38	Ref 3.04–9.51	Ref 5.773	Ref <0.001
Differentiations						
Poorly Moderately Highly	Ref −0.416 −2.218	Ref 0.232 0.739	Ref 0.66 0.11	Ref 0.42–1.04 0.03–0.46	Ref −1.791 −3.001	Ref 0.073 0.003
Diameter (cm)						
≤2 2–3 >3	Ref 0.648 0.954	Ref 0.261 0.272	Ref 1.91 2.60	Ref 1.15–3.19 1.52–4.42	Ref 2.479 3.503	Ref 0.013 <0.001
**PLR**	0.004	0.002	1.00	1.00–1.01	2.958	0.003

PLR, platelet-to-lymphocyte ratio.

### 3.4 Development and validation of the nomogram

**Figure 3:**
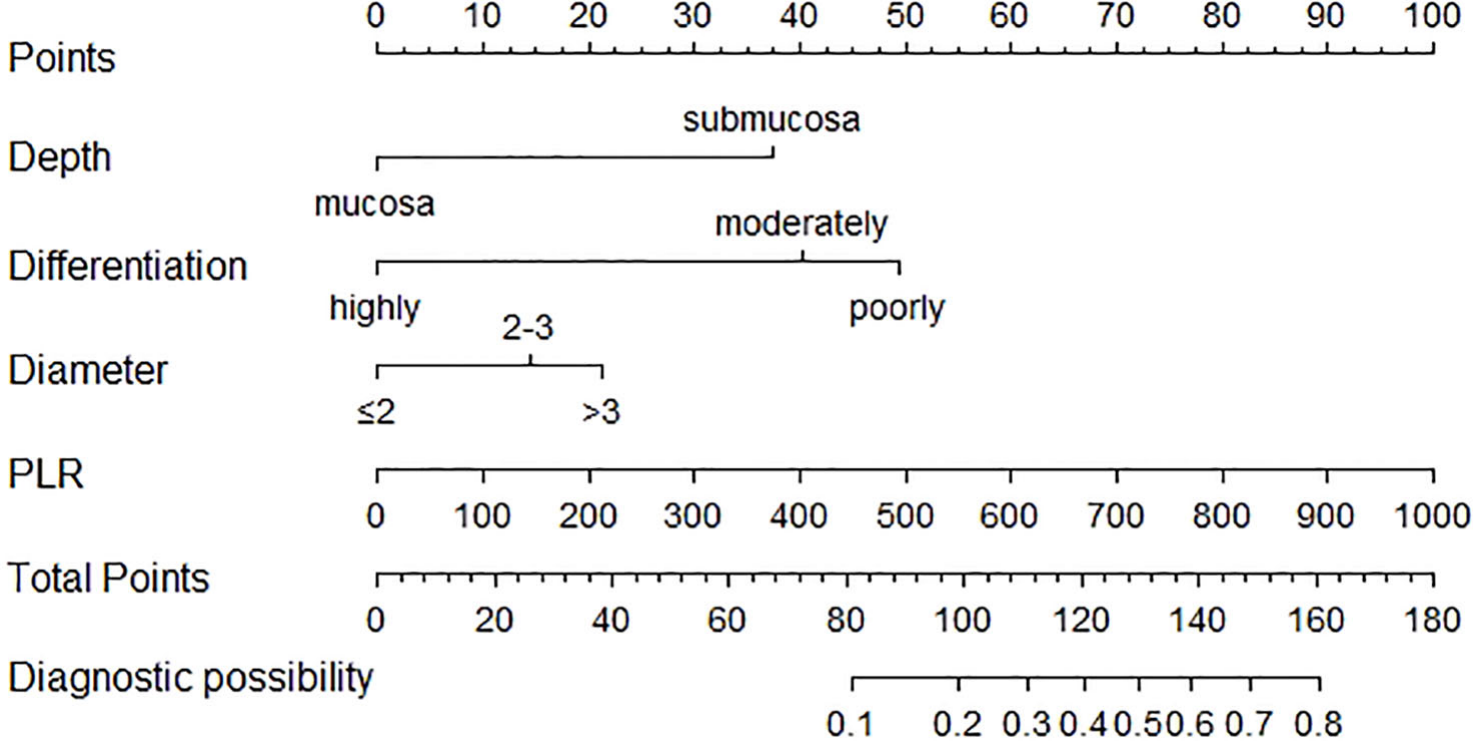
Nomogram for the prediction of LNM in EGC. PLR, platelet-to-lymphocyte ratio; LNM, lymph node metastasis; EGC, early gastric cancer.

**Figure 4:**
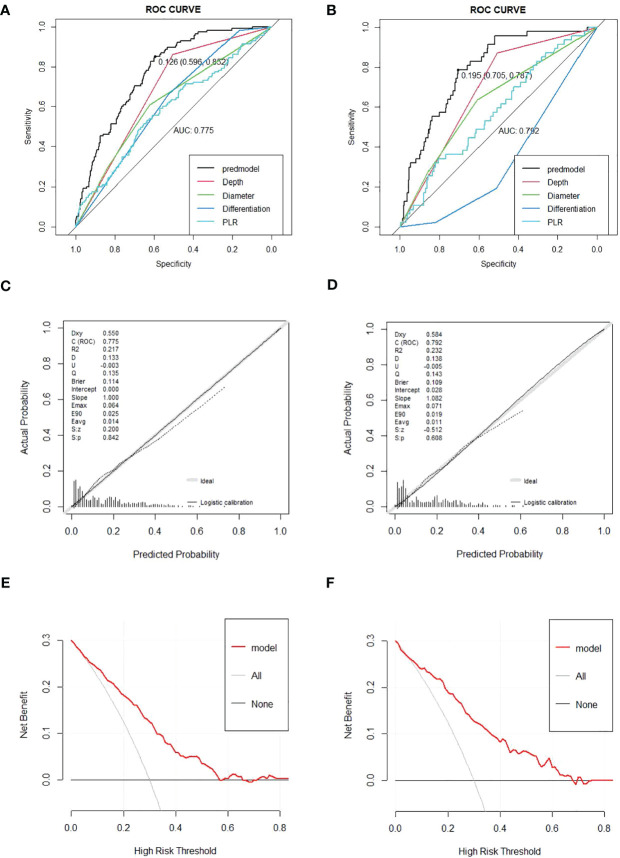
**(A, B)** Receiver operating characteristic (ROC) curves for the prediction of LNM in the training set and validation set. **(C, D)** Calibration curves in the training set and validation set. The x-axis represents the predicted probability from the nomogram, and the y-axis is the actual probability of LNM in GC patients. **(E, F)** Decision curve analysis (DCA) in the training set and validation set. The y-axis represents net benefits, calculated by subtracting the relative harm (false positives) from the benefits (true positives). The x-axis calculates the threshold probability. LNM, lymph node metastasis; GC, gastric cancer.

The nomogram was created depending on the impact of these variables on LNM (**Figure 3**). The line segments corresponding to each of the four variables are labeled with scales representing the range of the value. The Points (range from 0 to 100) represent the individual score for each variable at different values, and the sum of the individual scores of all the variables taken together is the Total Points (range from 0 to 180). By drawing a vertical line downward, we can find out the corresponding probability of lymph node metastasis in the patient. The AUC values were 0.775 (95%CI 0.734–0.816) and 0.792 (95%CI 0.729–0.855) for the training and validation groups, respectively. In the training set, the sensitivity was 0.852, and the specificity was 0.596. In the validation set, the sensitivity was 0.787, and the specificity was 0.705. The C-index was consistent with the AUC. Both calibration curves showed great consistency in the predictive and actual values. The Hosmer–Lemeshow (H-L) test was carried out in two cohorts, showing excellent performance with p-value >0.05 (0.684422, 0.7403046). Decision curve analysis was plotted in the training set and validation set to see a good clinical benefit (**Figure 4**). In the internal validation, 1,000 bootstraps showed that the accuracy of the model was 0.8515138.

## 4 Discussion

Currently, although image diagnostic technologies, such as computed tomography (CT), magnetic resonance imaging (MRI), and endoscopic ultrasonography (EUS), are able to detect the presence of enlarged regional lymph nodes, none of them is reliable for accurately judging the status of lymph nodes (29, 30). Therefore, new predictive methods are needed to estimate lymph node metastasis.

The problem has caused numerous discussions, while consensus has nearly been reached on the impact of factors like depth of invasion and degree of differentiation. When the lesion infiltrates into the submucosa, the risk of lymph node metastasis is significantly increased, which has been verified in many experiments (31–33) and is consistent with the results of our study. Meanwhile, poor differentiation is also reckoned as a higher risk factor for lymph node metastasis (6, 33–35). Additionally, research showed that LNM risk is proportional to tumor size (28, 36, 37). Other studies also proved that LNM is associated with gender, indicating that women with EGC have a higher risk of LNM (12, 28, 32, 38). However, corresponding conclusions cannot be drawn in our study.

In addition, the causal relationship between inflammation and cancer has been widely accepted. As is known to us, inflammation contributes to physiological and pathological processes, while the activation and directed migration of leukocytes from the venous system to sites of damage plays a crucial role, including neutrophils, monocytes, and eosinophils. At the same time, platelet activation and aggregation not only accelerate coagulation but also supplies the immediate area with large amounts of secreted proteins and alpha particles, all of which help initiate and accelerate the inflammatory response. Thus, PLR has been extensively examined in neoplastic diseases, while several studies have demonstrated the value of the level of PLR in evaluating the severity of systemic inflammation and prognosis. In our study, PLR is significantly correlated with the occurrence of LNM in patients with EGC, while in other studies, higher PLR were more likely to have LNM (39) and had decreased overall survival (OS) and disease-free survival (DFS) (23). However, the result of another study deviated from our results, which found that PLR had nothing to do with LNM as well as the prognosis of EGC patients (40). The opposite conclusions may be attributed to different participants. Nevertheless, our study had a larger sample size and conducted internal verification. So far, there have not been any predictive nomograms containing PLR as well as a good performance.

Even though previous studies have shown that lymphovascular invasion (LVI) is closely related to lymph node metastasis (31–33, 38, 41–43), we did not take it into our model on account of the fact that LVI cannot be confirmed before surgery. Moreover, some classic tumor markers like carbohydrate antigen 19-9 (CA19-9) (16, 17, 44, 45), HER-2 (46), and Ki-67 (17) are also included in several studies and have proven to be effective. What is novel is that Zhang, X., et al. proved the predictive value of fecal occult blood test (FOBT) (17) in patients after ESD, and the conclusion still needs additional proof. Nonetheless, since only a fraction of patients underwent the relative examination, we did not take the above variables into our study to avoid selection bias.

Our study confirmed the effectiveness of PLR in predicting LNM in patients with EGC before surgery and established a prediction nomogram based on preoperative data and pathological examination, although its utility in clinical practice needs to be further verified.

The study had several limitations. First, since it was a retrospective study, bias would inevitably exist due to the loss of data. Then, certain parameters such as tumor size and infiltration depth were confirmed by postoperative pathology, and deviations could appear between preoperative and postoperative biopsies. Finally, the detection of ulcers and the judgment of morphological features may vary slightly depending on the experience of different operating physicians.

## 5 Conclusion

The results of our study indicated that LNM is concerned with tumor size, depth of infiltration, degree of differentiation, and PLR. Aside from traditional pathological markers, the impact of PLR on patients deserves further exploration.

## Data availability statement

The original contributions presented in the study are included in the article/supplementary material. Further inquiries can be directed to the corresponding author.

## Ethics statement

The studies involving humans were approved by the Ethics Committee of the First Affiliated Hospital of Soochow University. The studies were conducted in accordance with the local legislation and institutional requirements. The ethics committee/institutional review board waived the requirement of written informed consent for participation from the participants or the participants’ legal guardians/next of kin because it was a retrospective analysis and informed consent statements from patients cannot be obtained.

## Author contributions

HW and WL conceived the study and drafted the manuscript. MY helped revise the manuscript critically. LL, SQ, and WX helped collect data, analyze the data, and design the study. All authors contributed to the article and approved the submitted version.

## References

[1] Sung H Ferlay J Siegel RL Laversanne M Soerjomataram I Jemal A. Global cancer statistics 2020: GLOBOCAN estimates of incidence and mortality worldwide for 36 cancers in 185 countries. CA: Cancer J Clin (2021) 71(3):209–49. doi: 10.3322/caac.21660 33538338

[2] Chen WQ Li H Sun KX Zheng RS Zhang SW Zeng HM. Report of cancer incidence and mortality in China, 2014. Zhonghua Zhong Liu Za Zhi (2018) 40(1):5–13. doi: 10.3760/cma.j.issn.0253-3766.2018.01.002 29365411

[3] Smyth EC Nilsson M Grabsch HI van Grieken NCT Lordick F. Gastric cancer. Lancet (2020) 396(10251):635–48. doi: 10.1016/S0140-6736(20)31288-5 32861308

[4] Japanese Gastric Cancer Association. Japanese classification of gastric carcinoma: 3rd English edition. Gastric Cancer (2011) 14(2):101–12. doi: 10.1007/s10120-011-0041-5 21573743

[5] Zheng Z Liu Y Bu Z Zhang L Li Z Du H. Prognostic role of lymph node metastasis in early gastric cancer. Chin J Cancer Res (2014) 26(2):192–9. doi: 10.3978/j.issn.1000-9604.2014.04.06 PMC4000899 24826060

[6] Lin JX Wang ZK Wang W Desiderio J Xie JW Wang JB. Risk factors of lymph node metastasis or lymphovascular invasion for early gastric cancer: a practical and effective predictive model based on international multicenter data. BMC cancer (2019) 19(1):1048. doi: 10.1186/s12885-019-6147-6 31694573 PMC6836519

[7] Ono H Yao K Fujishiro M Oda I Uedo N Nimura S. Guidelines for endoscopic submucosal dissection and endoscopic mucosal resection for early gastric cancer (second edition). Dig Endosc (2021) 33(1):4–20. doi: 10.1111/den.13883 33107115

[8] Kishida Y Takizawa K Kakushima N Kawata N Yoshida M Yabuuchi Y. Endoscopic submucosal dissection versus surgery in elderly patients with early gastric cancer of relative indication for endoscopic resection. Dig Endosc (2022) 34(3):497–507. doi: 10.1111/den.14105 34379850

[9] Ahn JY Kim YI Shin WG Yang HJ Nam SY Min BH. Comparison between endoscopic submucosal resection and surgery for the curative resection of undifferentiated-type early gastric cancer within expanded indications: a nationwide multi-center study. Gastric Cancer (2021) 24(3):731–43. doi: 10.1007/s10120-020-01140-x 33211219

[10] Lim JH Kim J Kim SG Chung H. Long-term clinical outcomes of endoscopic vs. surgical resection for early gastric cancer with undifferentiated histology. Surg endoscopy (2019) 33(11):3589–99. doi: 10.1007/s00464-018-06641-6 30604260

[11] Gockel I Hoffmeister A. Endoscopic or surgical resection for gastro-esophageal cancer. Dtsch Arztebl Int (2018) 115(31-32):513–9. doi: 10.3238/arztebl.2018.0513 PMC6131362 30149830

[12] Gu L Chen M Khadaroo PA Ma X Kong L Li X. A risk-scoring model for predicting lymph node metastasis in early gastric cancer patients: a retrospective study and external validation. J gastrointestinal Surg (2018) 22(9):1508–15. doi: 10.1007/s11605-018-3816-8 29845571

[13] Mu J Jia Z Yao W Song J Cao X Jiang J. Predicting lymph node metastasis in early gastric cancer patients: development and validation of a model. Future Oncol (London England) (2019) 15(31):3609–17. doi: 10.2217/fon-2019-0377 31517515

[14] Wang Z Liu J Luo Y Xu Y Liu X Wei L. Establishment and verification of a nomogram for predicting the risk of lymph node metastasis in early gastric cancer. Rev espanola enfermedades digestivas: organo oficial la Sociedad Espanola Patologia Digestiva (2021) 113(6):411–7. doi: 10.17235/reed.2020.7102/2020 33222482

[15] Sui W Chen Z Li C Chen P Song K Wei Z. Nomograms for predicting the lymph node metastasis in early gastric cancer by gender: A retrospective multicentric study. Front Oncol (2021) 11:616951. doi: 10.3389/fonc.2021.616951 34660252 PMC8511824

[16] Zhang M Ding C Xu L Feng S Ling Y Guo J. A nomogram to predict risk of lymph node metastasis in early gastric cancer. Sci Rep (2021) 11(1):22873. doi: 10.1038/s41598-021-02305-z 34819570 PMC8613278

[17] Zhang X Yang D Wei Z Yan R Zhang Z Huang H. Establishment of a nomogram for predicting lymph node metastasis in patients with early gastric cancer after endoscopic submucosal dissection. Front Oncol (2022) 12:898640. doi: 10.3389/fonc.2022.898640 36387114 PMC9651963

[18] Hatta W Gotoda T Oyama T Kawata N Takahashi A Yoshifuku Y. Is the eCura system useful for selecting patients who require radical surgery after noncurative endoscopic submucosal dissection for early gastric cancer? A comparative study. Gastric Cancer (2018) 21(3):481–9. doi: 10.1007/s10120-017-0769-7 28983696

[19] Hatta W Gotoda T Oyama T Kawata N Takahashi A Yoshifuku Y. A Scoring System to Stratify Curability after Endoscopic Submucosal Dissection for Early Gastric Cancer: “eCura system”. Am J Gastroenterol (2017) 112(6):874–81. doi: 10.1038/ajg.2017.95 28397873

[20] Ono H Yao K Fujishiro M Oda I Nimura S Yahagi N. Guidelines for endoscopic submucosal dissection and endoscopic mucosal resection for early gastric cancer. Dig Endosc (2016) 28(1):3–15. doi: 10.1111/den.12518 26234303

[21] Hatta W Gotoda T Koike T Masamune A. History and future perspectives in Japanese guidelines for endoscopic resection of early gastric cancer. Dig Endosc (2020) 32(2):180–90. doi: 10.1111/den.13531 31529716

[22] Zhang LX Wei ZJ Xu AM Zang JH. Can the neutrophil-lymphocyte ratio and platelet-lymphocyte ratio be beneficial in predicting lymph node metastasis and promising prognostic markers of gastric cancer patients? Tumor maker retrospective study. Int J Surg (London England) (2018) 56:320–7. doi: 10.1016/j.ijsu.2018.06.037 29969732

[23] Yun JM Chung MK Baek CH Son YI Ahn MJ Oh D. Prognostic significance of the post-treatment neutrophil-to-lymphocyte ratio in pharyngeal cancers treated with concurrent chemoradiotherapy. Cancers (2023) 15(4):1248. doi: 10.3390/cancers15041248 36831590 PMC9954210

[24] Yoshida K Yoshikawa N Shirakawa A Niimi K Suzuki S Kajiyama H. Prognostic value of neutrophil-to-lymphocyte ratio in early-stage ovarian clear-cell carcinoma. J Gynecol Oncol (2019) 30(6):e85. doi: 10.3802/jgo.2019.30.e85 31576683 PMC6779610

[25] Diem S Schmid S Krapf M Flatz L Born D Jochum W. Neutrophil-to-Lymphocyte ratio (NLR) and Platelet-to-Lymphocyte ratio (PLR) as prognostic markers in patients with non-small cell lung cancer (NSCLC) treated with nivolumab. Lung Cancer (2017) 111:176–81. doi: 10.1016/j.lungcan.2017.07.024 28838390

[26] Yodying H Matsuda A Miyashita M Matsumoto S Sakurazawa N Yamada M. Prognostic significance of neutrophil-to-lymphocyte ratio and platelet-to-lymphocyte ratio in oncologic outcomes of esophageal cancer: A systematic review and meta-analysis. Ann Surg Oncol (2016) 23(2):646–54. doi: 10.1245/s10434-015-4869-5 26416715

[27] The Paris endoscopic classification of superficial neoplastic lesions: esophagus, stomach, and colon: November 30 to December 1, 2002. Gastrointestinal endoscopy (2003) 58(6 Suppl):S3–43. doi: 10.1016/s0016-5107(03)02159-x 14652541

[28] Chen J Zhao G Wang Y. Analysis of lymph node metastasis in early gastric cancer: a single institutional experience from China. World J Surg Oncol (2020) 18(1):57. doi: 10.1186/s12957-020-01834-7 32197625 PMC7085136

[29] Wada T Yoshikawa T Kamiya A Date K Hayashi T Otsuki S. A nodal diagnosis by computed tomography is unreliable for patients who need additional gastrectomy after endoscopic submucosal dissection. Surg Today (2020) 50(9):1032–8. doi: 10.1007/s00595-020-01985-w 32130519

[30] Du M Chen L Cheng Y Wang Y Fan X Zhang Y. Tumor budding and other risk factors of lymph node metastasis in submucosal early gastric carcinoma: A multicenter clinicopathologic study in 621 radical gastrectomies of Chinese patients. Am J Surg pathol (2019) 43(8):1074–82. doi: 10.1097/PAS.0000000000001276 31094925

[31] Ren MH Qi XS Chu YN Yu YN Chen YQ Zhang P. Risk of lymph node metastasis and feasibility of endoscopic treatment in ulcerative early gastric cancer. Ann Surg Oncol (2021) 28(4):2407–17. doi: 10.1245/s10434-020-09153-7 PMC7940277 32975685

[32] Pyo JH Lee H Min BH Lee JH Choi MG Lee JH. Early gastric cancer with a mixed-type Lauren classification is more aggressive and exhibits greater lymph node metastasis. J gastroenterol (2017) 52(5):594–601. doi: 10.1007/s00535-016-1254-5 27590416

[33] Bausys R Bausys A Vysniauskaite I Maneikis K Klimas D Luksta M. Risk factors for lymph node metastasis in early gastric cancer patients: Report from Eastern Europe country- Lithuania. BMC surgery (2017) 17(1):108. doi: 10.1186/s12893-017-0304-0 29169358 PMC5701498

[34] Milhomem LM Milhomem-Cardoso DM da Mota OM Mota ED Kagan A Filho JBS. Risk of lymph node metastasis in early gastric cancer and indications for endoscopic resection: is it worth applying the east rules to the west? Surg endoscopy (2021) 35(8):4380–8. doi: 10.1007/s00464-020-07932-7 32880748

[35] Oh SY Lee KG Suh YS Kim MA Kong SH Lee HJ. Lymph node metastasis in mucosal gastric cancer: reappraisal of expanded indication of endoscopic submucosal dissection. Ann Surg (2017) 265(1):137–42. doi: 10.1097/SLA.0000000000001649 28009738

[36] Liang XQ Wang Z Li HT Ma G Yu WW Zhou HC. Indication for endoscopic treatment based on the risk of lymph node metastasis in patients with undifferentiated early gastric cancer. Asian J surgery (2020) 43(10):973–7. doi: 10.1016/j.asjsur.2019.12.002 31964584

[37] Chen L Wang YH Cheng YQ Du MZ Shi J Fan XS. Risk factors of lymph node metastasis in 1620 early gastric carcinoma radical resections in Jiangsu Province in China: A multicenter clinicopathological study. J digestive diseases (2017) 18(10):556–65. doi: 10.1111/1751-2980.12545 28949436

[38] Zhong Q Sun Q Xu GF Fan XQ Xu YY Liu F. Differential analysis of lymph node metastasis in histological mixed-type early gastric carcinoma in the mucosa and submucosa. World J gastroenterol (2018) 24(1):87–95. doi: 10.3748/wjg.v24.i1.87 29358885 PMC5757129

[39] Lou N Zhang L Chen XD Pang WY Arvine C Huang YP. A novel scoring system associating with preoperative platelet/lymphocyte and clinicopathologic features to predict lymph node metastasis in early gastric cancer. J Surg Res (2017) 209:153–61. doi: 10.1016/j.jss.2016.10.011 28032552

[40] Zhu GS Tian SB Wang H Ma MG Liu Y Du HS. Preoperative neutrophil lymphocyte ratio and platelet lymphocyte ratio cannot predict lymph node metastasis and prognosis in patients with early gastric cancer: a single institution investigation in China. Curr Med Sci (2018) 38(1):78–84. doi: 10.1007/s11596-018-1849-6 30074155

[41] Kang HJ Chung H Kim SG Kim J Kim JL Lee E. Synergistic effect of lymphatic invasion and venous invasion on the risk of lymph node metastasis in patients with non-curative endoscopic resection of early gastric cancer. J gastrointestinal Surg (2020) 24(7):1499–509. doi: 10.1007/s11605-019-04302-0 31313145

[42] Kim JY Kim CH Lee Y Lee JH Chae YS. Tumour infiltrating lymphocytes are predictors of lymph node metastasis in early gastric cancers. Pathology (2017) 49(6):589–95. doi: 10.1016/j.pathol.2017.06.003 28843920

[43] Huang Q Cheng Y Chen L Mingzhan D Wang Y Xu G. Low risk of lymph node metastasis in 495 early gastric cardiac carcinomas: a multicenter clinicopathologic study of 2101 radical gastrectomies for early gastric carcinoma. Modern Pathol (2018) 31(10):1599–607. doi: 10.1038/s41379-018-0063-1 29802360

[44] Yin XY Pang T Liu Y Cui HT Luo TH Lu ZM. Development and validation of a nomogram for preoperative prediction of lymph node metastasis in early gastric cancer. World J Surg Oncol (2020) 18(1):2. doi: 10.1186/s12957-019-1778-2 31898548 PMC6941310

[45] Liu Z Tian H Huang Y Liu Y Zou F Huang C. Construction of a nomogram for preoperative prediction of the risk of lymph node metastasis in early gastric cancer. Front surgery (2022) 9:986806. doi: 10.3389/fsurg.2022.986806 PMC9852636 36684356

[46] Mei Y Wang S Feng T Yan M Yuan F Zhu Z. Nomograms involving HER2 for predicting lymph node metastasis in early gastric cancer. Front Cell Dev Biol (2021) 9:781824. doi: 10.3389/fcell.2021.781824 35004681 PMC8740268

